# Zinc metalloprotease FgM35, which targets the wheat zinc-binding protein TaZnBP, contributes to the virulence of *Fusarium graminearum*

**DOI:** 10.1007/s44154-024-00171-z

**Published:** 2024-10-30

**Authors:** Xin-tong Wang, Kou-han Liu, Ying Li, Yan-yan Ren, Qiang Li, Bao-tong Wang

**Affiliations:** State Key Laboratory of Crop Stress Resistance and High-Efficiency Production, College of Plant Protection, Northwest A&F University, Yangling, Shannxi Province 712100 People’s Republic of China

**Keywords:** *Fusarium graminearum*, FgM35, Virulence, Plant immunology, Pathogen–Host

## Abstract

Metalloproteinases are ubiquitous in organisms. Most metalloproteinases secreted by pathogenic microorganisms are also called virulence factors, because they degrade proteins in the external tissues of the host, thereby reducing the host’s immunity and increasing its susceptibility to disease. Zinc metalloproteinase is one of the most common metalloproteinases. In our report, we studied the biological function of zinc metalloprotease FgM35 in *Fusarium graminearum* and the pathogen–host interaction during infection. We found that the asexual and sexual reproduction of the deletion mutant *ΔFgM35* were affected, as well as the tolerance of *F. graminearum* to metal stress. In addition, deletion of *FgM35* reduced the virulence of *F. graminearum*. The wheat target TaZnBP was screened using a wheat yeast cDNA library, and the interaction between FgM35 and TaZnBP was verified by HADDOCK molecular docking, yeast two-hybrid, Bi-FC, Luc, and Co-IP assays. The contribution of TaZnBP to plant immunity was also demonstrated. In summary, our work revealed the indispensable role of FgM35 in the reproductive process and the pathogenicity of *F. graminearum*, and it identified the interaction between FgM35 and TaZnBP as well as the function of TaZnBP. This provides a theoretical basis for further study of the function of metalloproteinases in pathogen–host interactions.

**Supplementary Information:**

The online version contains supplementary material available at 10.1007/s44154-024-00171-z.

## Introduction

Fusarium head blight (FHB) is one of the most serious diseases in wheat (Ma et al. 2020a, b). The disease is caused by a variety of *Fusarium* infections, among which *Fusarium graminearum* is the main pathogen (McMullen et al. 2012). FHB is greatly affected by climate, and as such it is more serious in warm and humid locations (Figueroa et al. 2018). FHB mainly damages wheat spikes, and it invades the plant at the base of wheat glumes during the flowering period. Brown disease spots form first, and then spread, resulting in ear rot and forming a pink mold layer. During the flowering period, the early infected spikes with spotted black asci can be seen when rainy weather occurs. The diseased panicles are significantly weaker than normal panicles in morphology, and grains may not develop in one or more glume, or even the whole panicle. Since the first report of FHB in the United Kingdom in 1884, the disease has spread to Asia, Europe, South America, and other parts of the world. At the end of the twentieth century, FHB was widespread in the United States, causing direct economic losses of up to 360 million dollars (Wilson et al. 2017). In addition, FHB produces a variety of toxins during the infection of host wheat grains by *F. graminearum*, the pathogen of FHB, significantly reduces the yield and the reduction of wheat quality; the most impactful of which are zearalenone (ZEA), deoxynivalenol (DON), and 3-acetyl deoxynivalenol (3-AcDON). The most common mycotoxin, DON, is one of the most dangerous toxins. It is also known as vomiting toxin, and has been listed as a third-level carcinogen by the European Union (Hu et al. 2020; Ma et al. 2020a, b). DON inhibits protein synthesis by interacting with protein synthesis-related factors in humans and animals, causing adverse reactions after ingestion such as vomiting, diarrhea, and nausea, which can lead to death in severe cases (Hou et al. 2023).

When *F. graminearum* invades the host plant, the pathogen secretes effector proteins into the plant that induce a series of susceptibility-resistance reactions. Therefore, the study of secreted proteins during infection can identify its pathogenic process, which helps to reveal the mechanism of interaction between pathogens and host plants (Xu et al. 2022). In general, further study on the mechanism of the *F. graminearum*-plant interaction is necessary for the developing new strategies for the prevention and control of FHB.

Metalloproteases are proteases that contain metal ions in the active center. Zinc metalloprotease is one of the most common metalloproteases. Because positively charged zinc ions have different oxidation states, it is easy to activate water molecules by electrons transformation of an unstable state. Jiang and Bond (1992) divided zinc metalloproteases into five families. Subsequently, Hooper (1994) divided the zinc metalloprotease superfamily into four families according to the different zinc-binding motifs in zinc metalloproteases: carboxypeptidase (HXXE), zinc protein (HEXXH), DD-carboxypeptidase (HXH), and anti-zinc protein (HXXEH). Most metalloproteinases secreted by pathogenic microorganisms are also called virulence factors because they degrade proteins in the external tissues of the host, thereby reducing the host’s immunity and increasing its susceptibility. For example, the metalloproteases Mrmep1 and Mrmep2 of *Metarhizium robertsii* regulate cell growth, spore production, and cell wall stability, and host immunity reduction (Zhou et al. 2018). The metalloprotease Cgf1, secreted by *Colletotrichum graminicola*, was used as a virulence factor to destroy host tissue (Sanz-Martin et al. 2016), and the M36 metalloprotease FgFly1 produced by *F. graminearum* was shown to be involved in the pathogenesis of *F. graminearum* (Wang et al. 2022). Zinc metalloproteases exist in different pathogenic microorganisms (Tallant et al. 2006). M35 (deuterium lyase) and M36 (fungal enzyme) metalloproteinases are two major zinc metalloproteinases secreted by pathogenic fungi. These two metalloproteases share a common zinc-binding motif, HEXXH, in which two histidine (H) residues act as the first and second zinc ligands, respectively (Rawlings et al. 2006). In addition to the HEXXH motif, M35 metalloprotease also contains the GTXDXXYG motif at the C-terminus, in which D (aspartic acid) is the third zinc ligand (Hori et al. 2001).

Metalloproteinases are considered to be important influencing factors or virulence factors in different pathogenic microorganisms. For example, the M35 metalloprotease RcMEP2, produced by *Rhizoctonia cerealis*, which causes rapid excessive accumulation of reactive oxygen species, induces cell death, inhibits the expression of host chitinase, and promotes pathogens infection (Pan et al. 2020). The entomopathogenic fungus *Metarhizium robertsii* secretes the metalloprotease MrM35-4, which is necessary for the virulence of fungi to insect hosts. It can promote the penetration of fungi into the cuticles of insects, inhibit the melanization activity of an insect epidermis, and also inhibit the antifungal genes expression of an insect to overcome the immune defense responses (Huang et al. 2020). FocM35_1, a metalloprotease effector produced by *Fusarium oxysporum*, which reduce plant chitinase activity, inhibit INF1-induced hypersensitivity (HR), and increase its toxicity by inhibiting host immunity (Zhang et al. 2021). In pathogenic bacteria *Aeromonas salmonicida* of fish, AsaP1 has indisputable bacterial virulence (Arnadottir et al. 2009).

At present, the function of M35 metalloproteinase is less studied in fungi, and the research mainly focuses on how it reduces host immunity as a virulence factor. However, the function of M35 metalloproteinase in *F. graminearum* has not been studied. In this study, we explored the function of FgM35 in *F. graminearum* and its effect on host immunity, which provide a scientific basis for the study of encoding M35 metalloproteinase in *F. graminearum* and a new theoretical basis for the prevention and control of FHB.

## Results

### Bioinformatics analysis of FgM35 and its expression level during infection

**Fig. 1:**
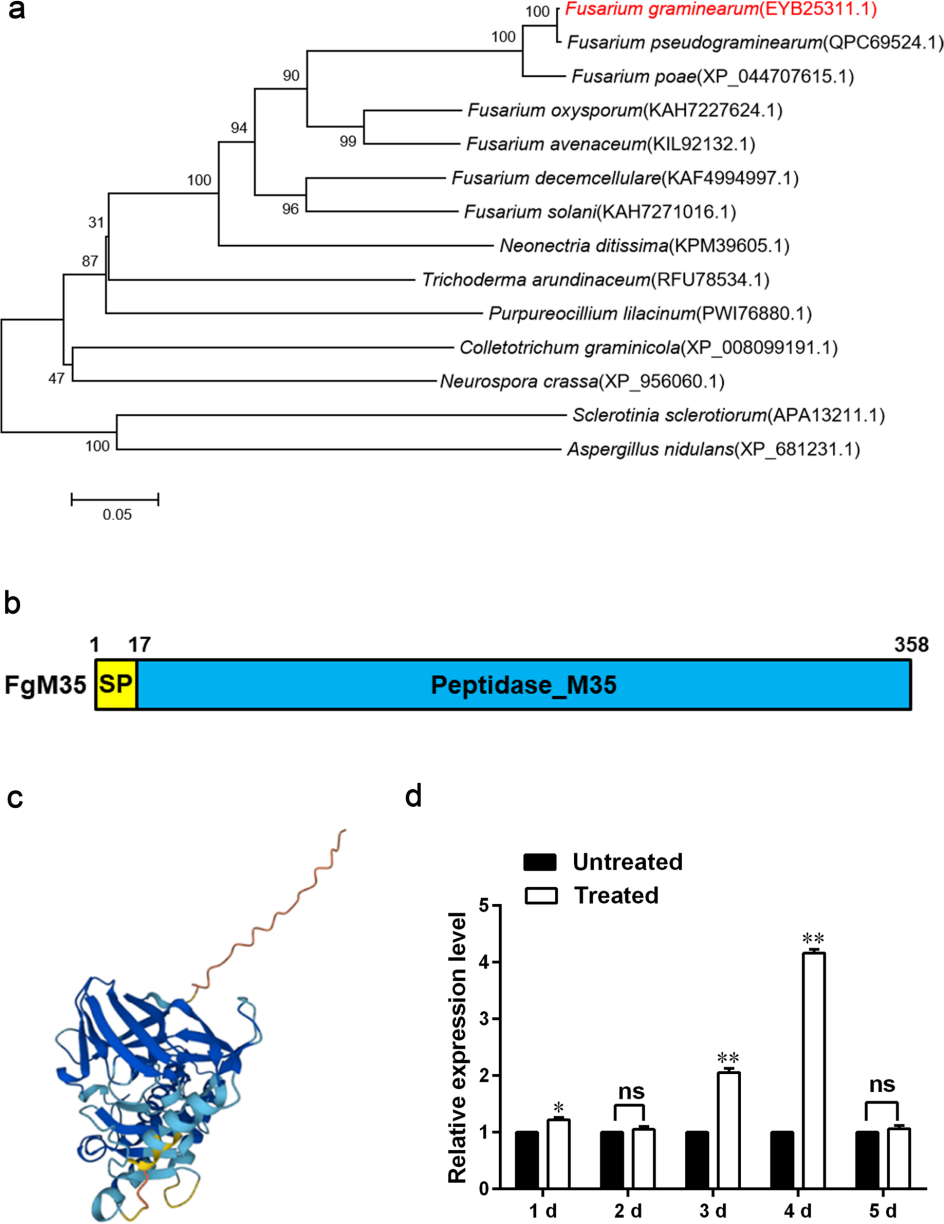
Phylogenetic tree and domain analysis of M35 homologs in different fungi constructed using MEGA5. **a**, Minimum evolution tree based on full-length sequence alignment of M35 homologs from different fungi. The tree was generated with Mega5 software using the minimum evolution algorithm with 1000 bootstrap replicates. The scale represents the number of substitutions per site. The numbers next to the species’ names correspond to the protein accession numbers in NCBI. **b**, Domain prediction of FgM35 using HMMER. **c**, Three-dimensional structure prediction of FgM35 protein by Alphafold2. **d**, Expression analysis of *FgM35* at different stages of wheat infection (Untreated: expression level of genes at different stages of uninfected wheat; Treated: expression level of genes at different stages of wheat infection). The experiment was repeated three times, and the average values and standard deviations were calculated based on the three repetitions. Values with the same letters at the end are not significantly different (*P* < 0.05)

The FgM35 protein sequence was compared with other filamentous fungal protein sequences through the NCBI website, and a phylogenetic tree was made using MEGA5 software (version 5.0, Mega Limited, Auckland, New Zealand). The results were shown in Fig. 1a. It was found that there were genes with high homology to FgM35 in *Fusarium pseudograminearum*, *Fusarium poae*, *F. oxysporum,* and *Fusarium avenaceum*, and these genes also encoded M35 metalloproteinase. Combined with phylogenetic tree analysis, *FgM35* was highly conserved in Fusarium.

Using the NCBI database (https://www.ncbi.nlm.nih.gov/), we found that the total length of the *FgM35* (FGSG_08289) gene was 1266 bp, containing introns and encoding 358 amino acids; its isoelectric point was 4.85, its protein molecular weight was 38.19 kDa, and it was located on chromosome 2 at 2359107–2360770 bp. The structure of the FgM35 protein was analyzed using HMMER (http://www.hmmer.org/) and SignalP (https://services.healthtech.dtu.dk/services/SignalP-5.0/). The results showed that FgM35 encoded M35 metalloproteinase and contained two conserved structural motifs, HEXXH and GTXDXXYG at the C-terminus. A signal peptide encoding 17 amino acids was predicted to be at the N-terminus, as shown in Fig. 1b. The three-dimensional structure of the FgM35 protein was predicted by the AlphaFold2 protein structure database (https://alphafold.ebi.ac.uk/), as shown in Fig. 1c.

To clarify the expression level of *FgM35* in different stages of infection, the RNA of wheat ears infected with wild-type strain PH-1 for 1–5 d was extracted, respectively. The untreated group was cultured with wild-type PH-1 for 1–5 d. The RNA was reversed into cDNA and determined by qRT-PCR. As shown in Fig. 1d, *FgM35* was upregulated to varying degrees at 1 d, 3 d, and 4 d after infection, and the expression multiple was the highest at 4 d, where the expression was upregulated by about four times. The results showed that *FgM35* may have played a role in the interaction between *F. graminearum* and host wheat.

### ΔFgM35 affected the growth rate of *F*. *graminearum* but did not affect the hyphal edge bifurcation

In the previous experiment, we obtained and validated three phenotypically identical knockout transformants of *FgM35* (*∆FgM35*-1, *∆FgM35*-2, *∆FgM35*-3) (Figs. S1 and S2) and selected one of the knockout transformants for complementation to obtain a complemented strain (*∆FgM35*-C).

**Fig. 2:**
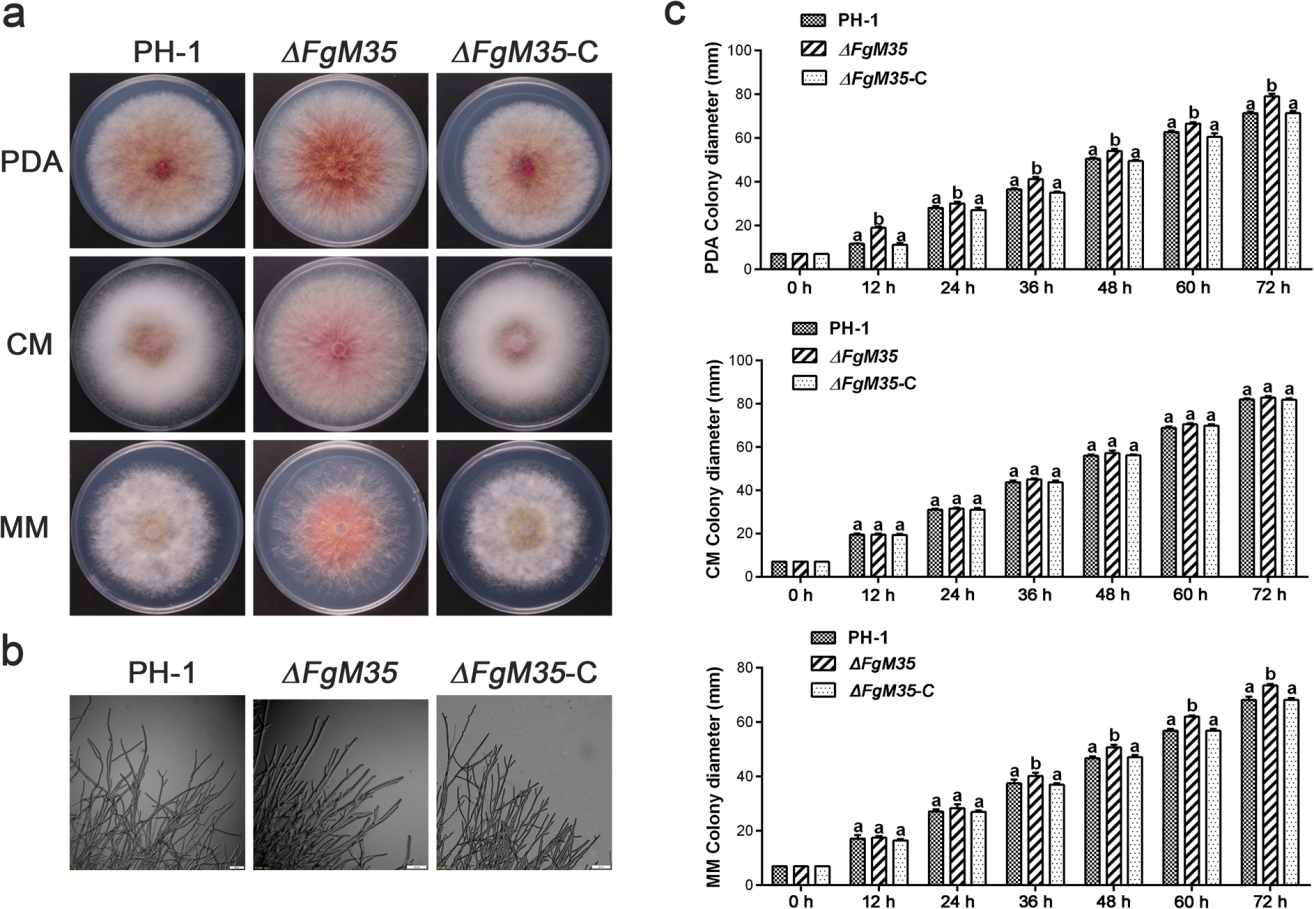
*ΔFgM35* impact on nutritional growth of *F. graminearum*. **a**, Wild-type strain PH-1, mutant strain *ΔFgM35*, and complemented strain *ΔFgM35*-C were inoculated on potato dextrose agar (PDA), complete medium (CM), and minimal medium (MM). Photographs were taken after three days of growth at 25 °C. **b**, Wild-type strain PH-1, mutant strain *ΔFgM35*, and complemented strain *ΔFgM35*-C were observed for hyphal branching at the edge of PDA-coated slides using an Olympus microscope after 24 h of growth (bar = 50 μm). **c**, Colony diameter was measured using the crossover method every 12 h, and measurements were taken continuously for three days. The experiment was repeated three times, and the average values and standard deviations were calculated based on the three repetitions. Values with the same letters at the end are not significantly different (*P* < 0.05)

Using an aseptic puncher, tender fungal discs were inoculated on PDA, CM, and MM media from the edge of freshly activated wild-type, mutant, and complemented strain colonies. They were then incubated at a constant temperature of 25℃, and the diameter of the colonies was measured every 12 h. After three days of cultivation, the morphology of the colonies was observed and photographed. As depicted in Fig. 2a, we discerned no significant aberrations in the colony morphology of the mutant *∆FgM35* strain in comparison with the wild-type PH-1 and complemented strain *∆FgM35*-C. The assessment of colony diameters revealed that the absence of *FgM35* slightly accelerated the growth rate of *F. graminearum* on PDA and MM media compared to rates of the wild-type and complemented strains. However, there was no significant perturbation in the growth rate of *∆FgM35* on the CM medium as compared to the rates of the wild-type and complemented strains, as shown in Fig. 2c. These findings suggested that *FgM35* minimally impacted the nutritional growth of *F. graminearum*.

Newly activated wild-type, mutant, and complemented strains were inoculated onto the outer surface of glass slides coated with PDA medium. The inoculated slides were then incubated at a constant temperature of 25 °C until hyphal growth extended onto the glass slide. Microscopic examination was conducted to observe the hyphal edges. As depicted in Fig. 2b, there were no apparent differences observed in terms of the quantity or density of hyphal branching between the mutant strain *∆FgM35* and the wild-type PH-1 and complemented strain *∆FgM35*-C. These results indicated that *FgM35* did not participate in the regulation of hyphal edge branching in *F. graminearum*.

### FgM35 had an effect on asexual/sexual reproduction of *F. graminearum*

**Fig. 3:**
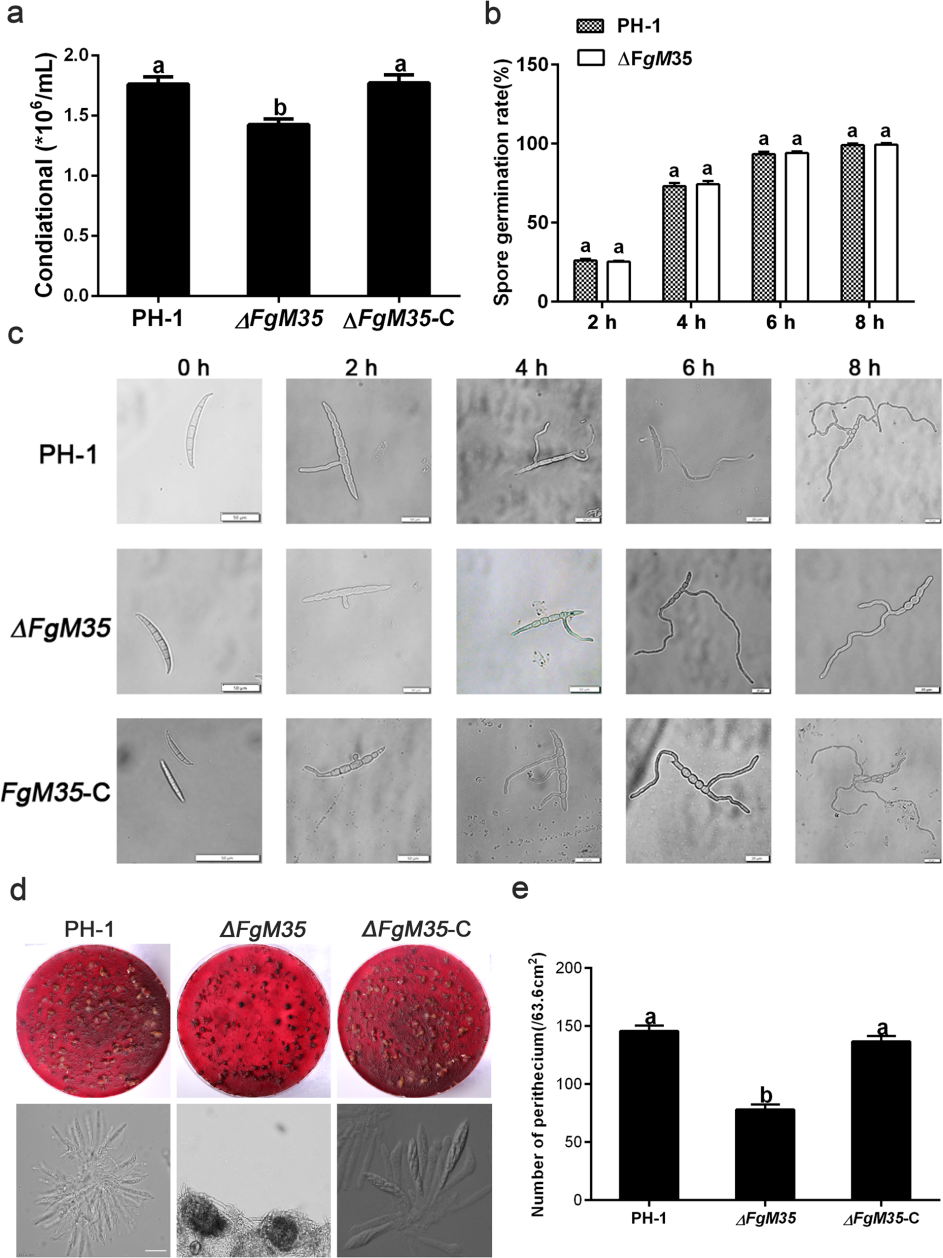
The impact of *ΔFgM35* on asexual/sexual reproduction in *F. graminearum*. **a**, Wild-type strain PH-1, mutant strain *ΔFgM35*, and complemented strain *ΔFgM35*-C were cultured on CMC medium at 25 °C and 180 rpm for three days, and the number of conidia in 10 uL of liquid was examined using a hemocytometer. **b**, All strains were cultured in YEPD liquid medium at 25 °C and 180 rpm, and the conidial germination rate was measured at 2 h, 4 h, 6 h, and 8 h. **c**, Morphology of conidia from wild-type strain PH-1, mutant strain *ΔFgM35*, and complemented strain *ΔFgM35*-C was observed under a microscope at 0 h, 2 h, 4 h, 6 h, and 8 h (bar = 50 μm). **d**, All strains were inoculated on carrot agar and induced for sexual reproduction under a 12 h dark: 12 h light cycle for 15 days. The presence of ascospores was observed by crushing the perithecia using a microscope (bar = 50 μm). **e**, The number of perithecia produced on carrot agar by all strains was counted. The experiment was repeated three times. The mean and standard deviation were calculated from three replicates. Values with different letters indicate significant differences (*P* < 0.05)

Five fresh agar plates each of wild-type, mutant, and complemented strains were inoculated in 30 mL of CMC medium, then incubated at 25 °C and 180 rpm for three days. Afterward, the spore suspension was filtered and 10 μL was pipetted onto a hemocytometer to count the number of conidia. As shown in Fig. 3a, compared to results of the wild-type strain PH-1, the ΔFgM35 mutant exhibited reduced sporulation, while the complemented strain ΔFgM35-C restored sporulation levels to that of PH-1. These results indicated that the mutation in ΔFgM35 affected the sporulation of *F. graminearum* conidia. Microscopic examination of conidial morphology of PH-1 and mutant *∆FgM35* at 0 h, 2 h, 4 h, 6 h, and 8 h in YEPD shaking cultures revealed no significant differences between the mutant and wild-type strains (Fig. 3c). Additionally, the germination rate of conidia was evaluated by randomly selecting 50 conidia at each time point, and the results showed that *∆FgM35* did not affect conidial germination in *F. graminearum* (Fig. 3b).

*F. graminearum* primarily overwinters on infected crop residue in the form of perithecia. When weather conditions are favorable, perithecia release ascospores, which dispersed onto wheat plants via wind or rainwater, leading to infection. Therefore, the quantity of perithecia may influence the severity of FHB in wheat (Ni et al. 2011). To assess perithecia production, wild-type strain PH-1, mutant *∆FgM35*, and complemented strain *∆FgM35*-C were inoculated on carrot agar medium, and perithecia formation was induced by a 12-h dark: 12-h light period. The number of perithecia was quantified, and compared to results of the wild-type and complemented strains, the mutant *∆FgM35* displayed a significant decrease in perithecia production (Fig. 3e). Microscopic examination of the perithecia revealed that the mutant *∆FgM35* failed to release asci upon squeezing, while the wild-type and complemented strains were capable of releasing asci (Fig. 3d). Thus, results suggested that *FgM35* influenced sexual reproduction in *F. graminearum.*

### FgM35 affects the response of *F. graminearum* to external stress

**Fig. 4:**
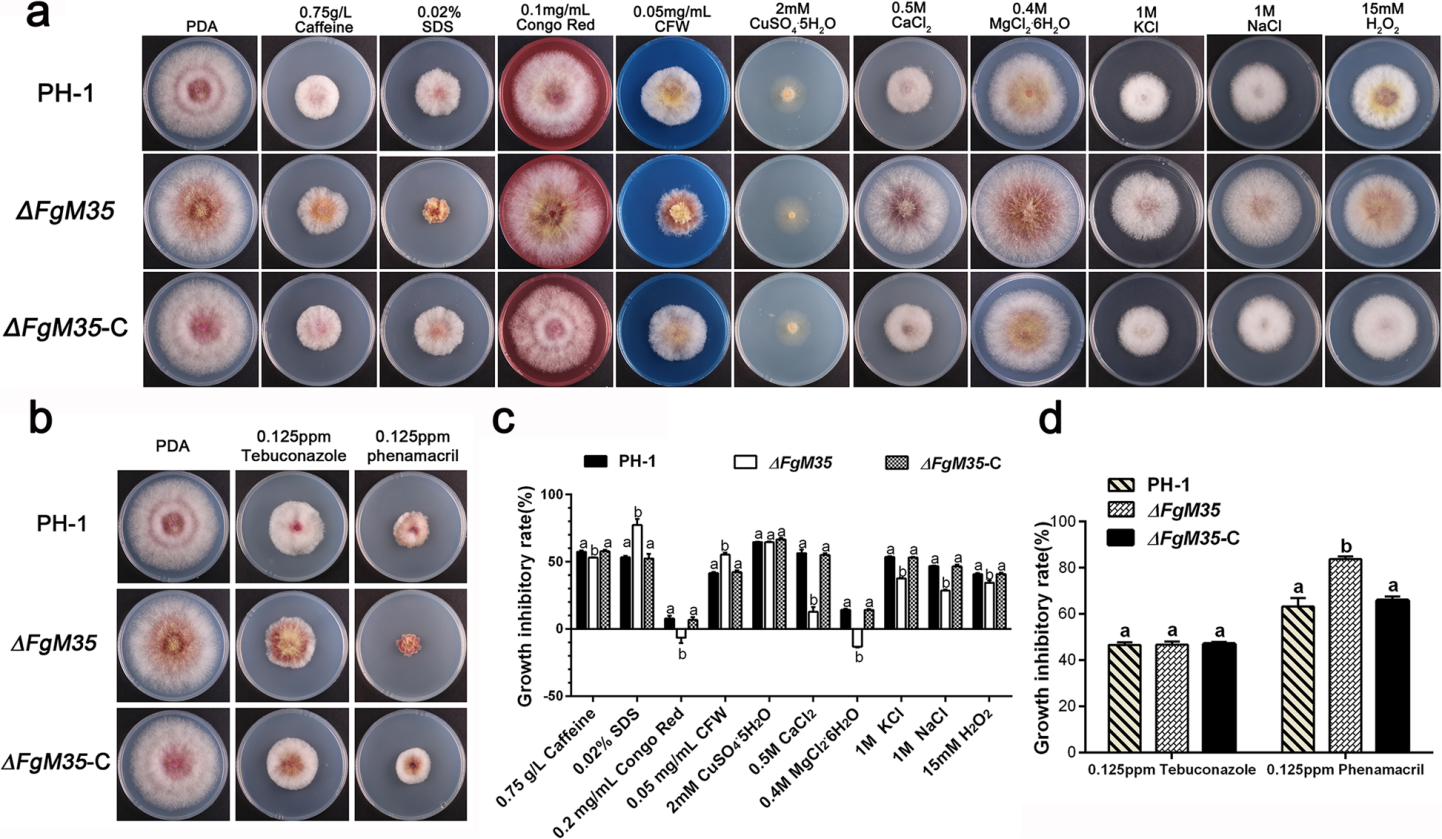
Sensitivity of the wild-type strain PH-1, mutant *ΔFgM35*, and complemented strain *ΔFgM35*-C to stress conditions. **a**, Sensitivity of all strains to osmotic, oxidative, metal ion, and cell wall stresses. Mycelial growth inhibition was evaluated after three days of stress cultivation. **b**, Sensitivity of all strains to fungicides. Mycelial growth inhibition was evaluated after three days of fungicide cultivation. **c**, Statistical analysis of the inhibition rates of all strains under osmotic, oxidative, metal ion, and cell wall stress conditions. **d**, Statistical analysis of the inhibition rates of all strains under fungicide stress. Mycelial growth inhibition rate (MGIR) was calculated as MGIR = [(N–C)/C] × 100, where C is the diameter of the control colony and N is the diameter of the treated colony. Mean values and standard deviations were calculated from three replicates. Values labeled with the same letter do not represent significant differences (*P* < 0.05)

As an integral component of the disease triangle, the external environment plays a crucial role in the process of pathogen invasion. To investigate the impact of *ΔFgM35* on *F. graminearum*’s response to external stressors, sensitivity experiments were conducted on factors such as cell wall stress, metal ions, fungicides, osmotic stress, and oxidative stress. Wild-type strain PH-1, mutant strain Δ*FgM35*, and complemented strain *ΔFgM35*-C were inoculated onto PDA plates containing 0.75 g/L caffeine, 0.02% SDS, 0.2 mg/mL Congo red, 0.05 mg/mL CFW, 2 mM CuSO_4_·5H_2_O, 0.5 M CaCl_2_, 0.4 M MgCl_2_·6H_2_O, 1 M KCl, 1 M NaCl, 15 mM H_2_O_2_, 0.125 ppm tebuconazole, and 0.125 ppm phenamacril, and were then incubated at 25℃ for three days for phenotypic observation (Fig. 4a). The growth inhibition rates were calculated.

As depicted in Fig. 4c, the analysis of growth inhibition rates revealed that under the pressure stress of 0.02% SDS and 0.05 mg/mL CFW, the mutant *∆FgM35* exhibited significantly higher growth inhibition rates compared to those of the wild-type PH-1 and complemented strain *∆FgM35*-C, indicating an enhanced sensitivity and weakened tolerance. This suggested that *FgM35* may play a crucial role in regulating the stability of *F. graminearum* cell wall in cereal crops. To further elucidate the sensitivity of mutant *∆FgM35* to metal ions, growth inhibition rates were calculated. These demonstrated that the mutant *∆FgM35* exhibited reduced growth inhibition rates in response to Mg^2+^ and Ca^2+^ compared to those of the wild-type PH-1 and complemented strain *∆FgM35*-C, indicating a decreased sensitivity and enhanced tolerance. Subsequently, sensitivity experiments were conducted to evaluate the mutant *∆FgM35*’s response to osmotic stress and oxidative stress. The results revealed that the mutant *∆FgM35* displayed decreased sensitivity to osmotic stress factors KCl and NaCl, as well as the oxidative stress inducer H_2_O_2_, thereby indicating an enhanced tolerance. These findings implied that *FgM35* may have been involved in regulating the response of *F. graminearum* to external osmotic stress and oxidative stress.

Tebuconazole and phenamacril are the main fungicides used to control wheat FHB. Tebuconazole, a triazole fungicide, inhibits the biosynthesis of ergosterol, a crucial component of fungal cell membranes, leading to the disruption of membrane structures within the cells and inhibition of fungal growth, thereby reducing pathogenicity (Menniti et al. 2003). Phenamacril inhibits the ATPase activity by targeting the muscle protein of *F. graminearum*, thereby interfering with normal cellular activities and achieving effects control (Tang et al. 2018). As shown in Fig. 4b, the response of the *F. graminearum* mutant strain *∆FgM35* to the fungicides tebuconazole and phenamacril was investigated and growth inhibition rates were calculated (Fig. 4d). Preliminary analysis indicated that the *∆FgM35* mutant exhibited increased sensitivity and decreased tolerance to phenamacril compared to those of the wild-type PH-1 and the complemented strain *∆FgM35*-C. However, there was no significant difference in sensitivity to tebuconazole. Therefore, it could be inferred that FgM35 may have been involved in the mechanism of action of phenamacril in *F. graminearum*.

### ∆FgM35 reduced the virulence of *F. graminearum*

**Fig. 5:**
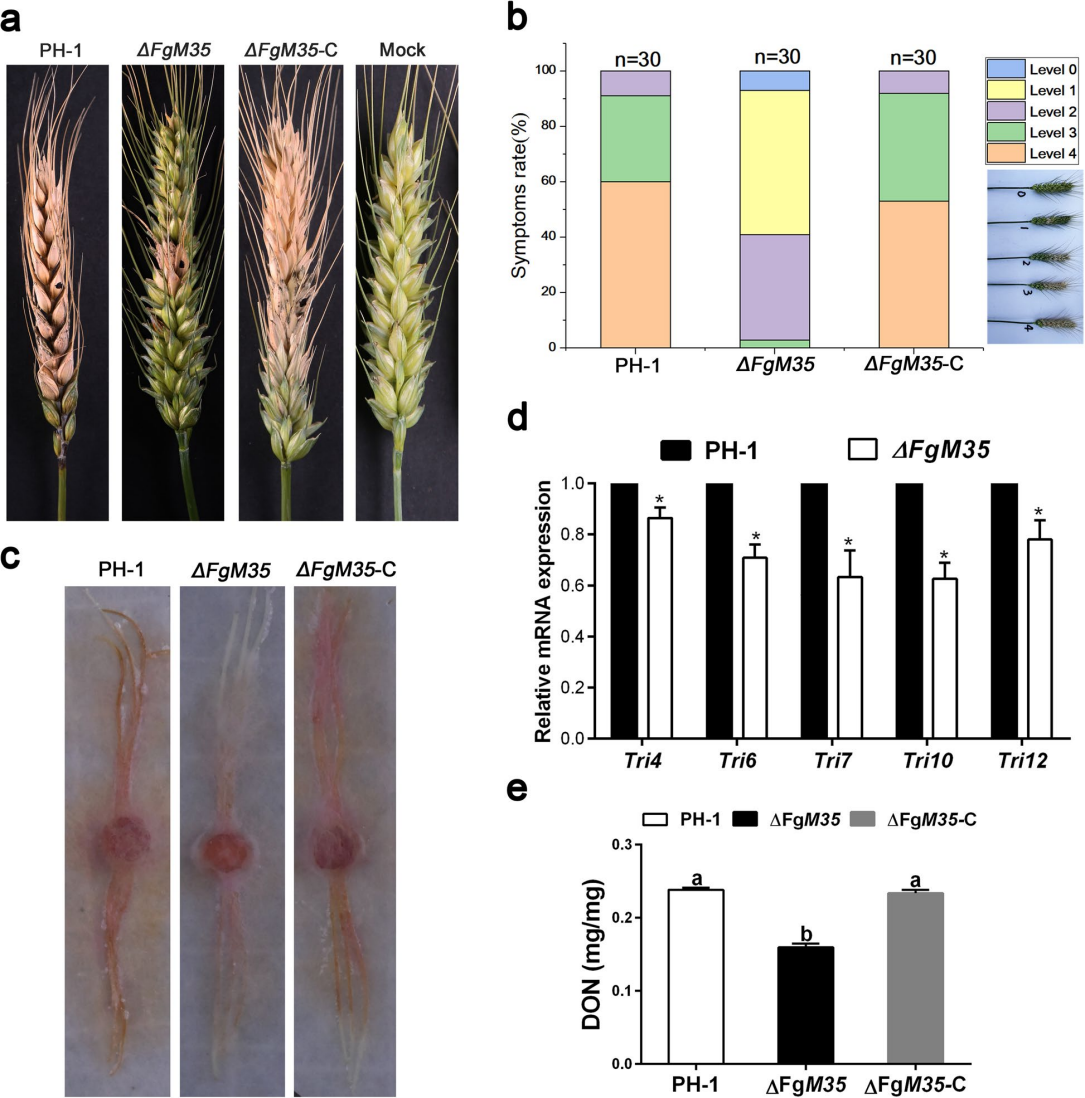
Contribution of the mutant strain *ΔFgM35* to pathogenicity and DON production. **a**, Inoculation of wheat spikes (Xiaoyan 22) with wild-type strain PH-1, mutant strain *ΔFgM35*, and complemented strain *ΔFgM35*-C, followed by photography 15 days post-inoculation. **b**, Disease severity rating of all strains in the field after wheat infection. **c**, Activation of all strains with fresh mycelial plugs inoculated onto maize silk and maintained at 25 °C under humid conditions, followed by photography four days post-inoculation. **d**, RNA extraction from wheat plants infected with wild-type strain PH-1 and mutant strain *ΔFgM35* in the field to measure the expression levels of toxin-related genes. The experiment was repeated three times, and the mean and standard deviation were calculated based on three replicates. Values with different letters at the end indicate significant differences (*P* < 0.05). **e**, Measurement of DON content in infected wheat spikes using an ELISA kit (Jiangsu Enzyme Immunoassay Co., Ltd., China) after field inoculation with all strains. The experiment was repeated three times, and the mean and standard deviation were calculated based on three replicates. Values with different letters at the end indicate significant differences (*P < *0.05)

The spore suspensions of each fungal strain were inoculated into the middle part of wheat spikes during the heading and flowering stages with 30 spikes per strain. Disease development was observed after two weeks. The results showed that the wild type and complemented strains resulted in extensive infection spreading from the inoculation site to most of the spikes, while the mutant strain *∆FgM35* showed infection symptoms only in a few small spikes near the inoculation site without significant spreading (Fig. 5a). The number of plants with severity grade 1 infection was higher in the plants inoculated with the mutant strain *∆FgM35* compared to other strains (Fig. 5b). Indoor silk infection experiments on corn showed similar results to the field pathogenicity tests (Fig. 5c). Therefore, it could be concluded that the pathogenicity of the mutant strain *∆FgM35* had been reduced.

The mycotoxin DON is an important virulence factor of *F. graminearum*, causing yield loss and affecting protein synthesis, leading to human and animals toxicity. The DON content were measured in the wheat grains infected by the wild-type strain PH-1, the mutant strain *∆FgM35*, and the complemented strain *∆FgM35*-C, respectively. The results showed a reduction in DON content in the grains infected by the mutant strain *∆FgM35* (Fig. 5e).

The expression of the *Tri* gene cluster plays a crucial role in the DON biosynthesis process. To further confirm the effect of F*gM35* on DON content, the expression levels of the *Tri* gene cluster in the wild-type strain PH-1 and the mutant strain *∆FgM35* were measured using qRT-PCR (Fig. 5d). The results showed downregulation of *Tri4*, *Tri6*, *Tri7*, *Tri10*, and *Tri12* genes in the mutant strain *∆FgM35* compared to the wild-type strain PH-1. Therefore, it could be concluded that *FgM35* in *F. graminearum* regulated the biosynthesis of DON by influencing the expression levels of the *Tri* genes and was essential for DON biosynthesis.

### FgM35 had secretory function and could inhibit INF1-induced hypersensitivity

**Fig. 6:**
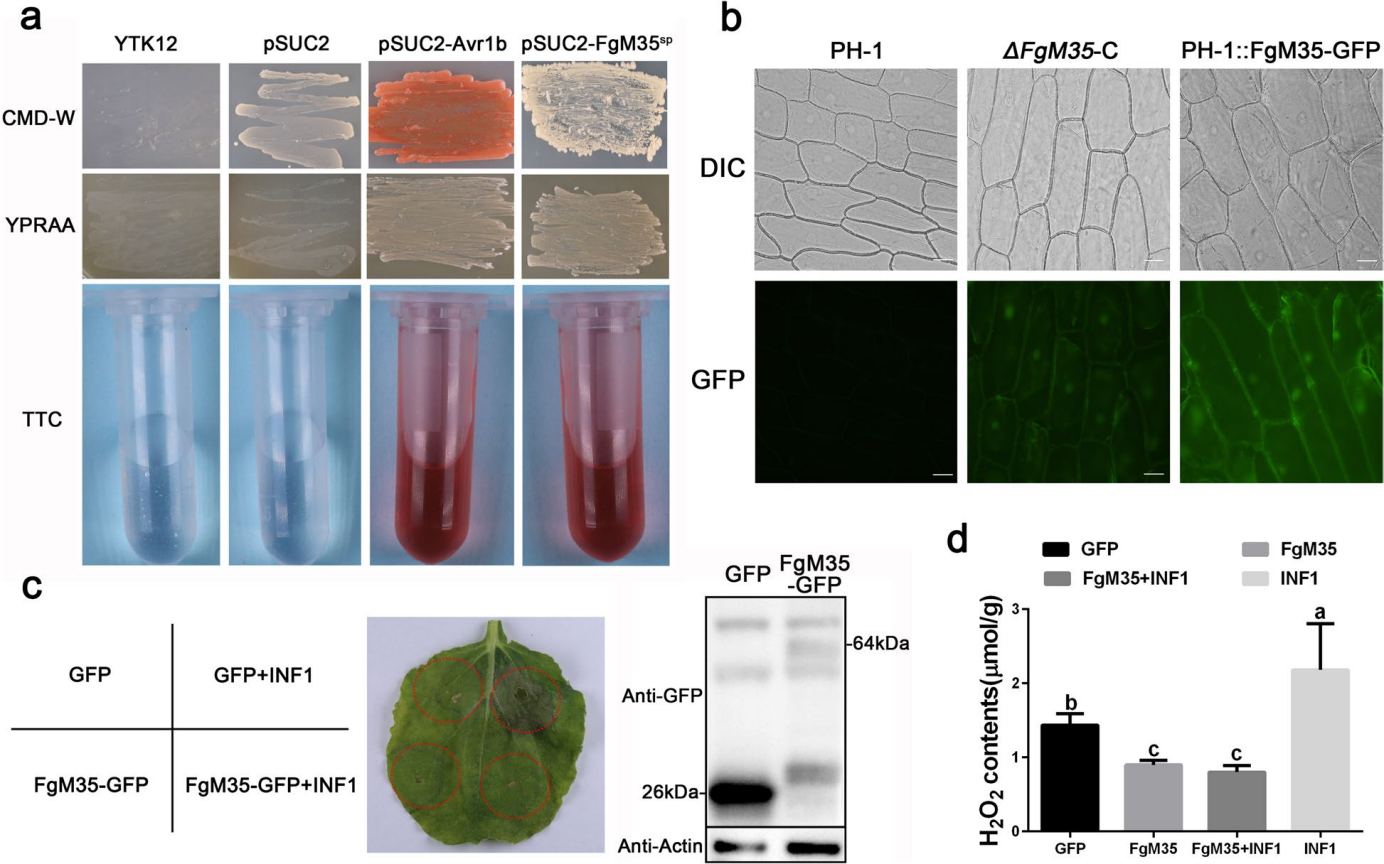
Validation of the signal peptide secretion function of FgM35 and its influence on reactive oxygen species (ROS) in *N. benthamiana*. **a**, The yeast YTK12 strain carrying the FgM35 signal peptide fragment fused in the pSUC2 vector was able to grow in both the CMD-W and YPRAA media and induced the TTC red reaction. **b**, FgM35-GFP fusion protein expressed in *ΔFgM35*-C strain and PH-1::*FgM35*-GFP strain of *F. graminearum*, respectively, showed green fluorescence signals in the onion epidermal cells after infection at 25 °C, 180 rpm for three days in CMC medium (bars = 50 μm). **c**, FgM35 suppressed the hypersensitive response induced by INF1 in *N. benthamiana*. The right panel shows a photograph taken after five days of infection with INF1, accompanied by Western blot analysis of GFP and FgM35-GFP protein expression levels. **d**, H_2_O_2_ content was measured in *N. benthamiana* leaves at 2 d post-infiltration with *A. tumefaciens* containing GFP, FgM35, FgM35 + INF1, and INF1. Error bars represent standard deviations from three repeated experiments. The same letter on the bars for each treatment indicates no significant difference (*P* = 0.05)

To ascertain whether FgM35 was a secreted protein, the signal peptide of FgM35 predicted by SignalP was functionally validated using the principles of the yeast secretion system. In this experiment, soybean *Phytophthora sojae* avirulence protein Avr1b, which has been proven to have secretion capacity, was used as a positive control, while the pSUC2 empty vector served as the negative control. As seen in Fig. 6a, it was concluded that the yeast strain carrying the fusion gene containing the FgM35 signal peptide was able to grow on CMD-W medium, indicating the successful transformation of the vector into the YTK12 strain. Furthermore, this fusion yeast strain exhibited similar characteristics to the positive control, as it could grow on YPRAA medium and turn the TTC solution red. Thus, it could be inferred that the FgM35 signal peptide possessed secretion capacity.

To further elucidate whether FgM35 could be secreted into the cytoplasm, spore suspensions of the complemented strain *ΔFgM35*-C and the overexpression strain PH-1::*FgM35* were used to invade onion epidermal cells. After three days of invasion and observation under a fluorescence microscope, fluorescence signals were detected in both the onion epidermal cells invaded by the complemented strain *ΔFgM35*-C and those invaded by the overexpression strain PH-1::*FgM35* (Fig. 6b), proved that FgM35 could be secreted into the cytoplasm.

INF1 triggers a hypersensitive response (HR) in plants, characterized by cell death in *Nicotiana benthamiana*. To investigate the effect of the FgM35 protein in plant immunity, FgM35 was fused to the pBIN-eGFP vector and transiently expressed in *N. benthamiana*. After 20 h, INF1 was injected into the same location, and leaf necrosis was observed 3–5 days later. Western blot analysis was employed to examine the expression levels of eGFP and eGFP-FgM35. As shown in Fig. 6c, a single injection of pBIN-eGFP or pBIN-eGFP-FgM35 did not induce cell death in *N. benthamiana*, while co-injection of pBIN-eGFP and INF1 resulted in cell death. However, co-injection of INF1 and pBIN-Egfp-FgM35 did not induce cell death, suggesting that FgM35 could suppress INF1-induced HR, thereby inhibiting plant immunity. We assessed the levels of reactive oxygen species (ROS) in *N. benthamiana* leaves after refractometer treatment, and the results in Fig. 6d clearly indicate a significant reduction in ROS levels compared to controls pBIN-eGFP + INF1 and INF1.

### TaZnBP enhanced plant immunity and interacted with FgM35

**Fig. 7:**
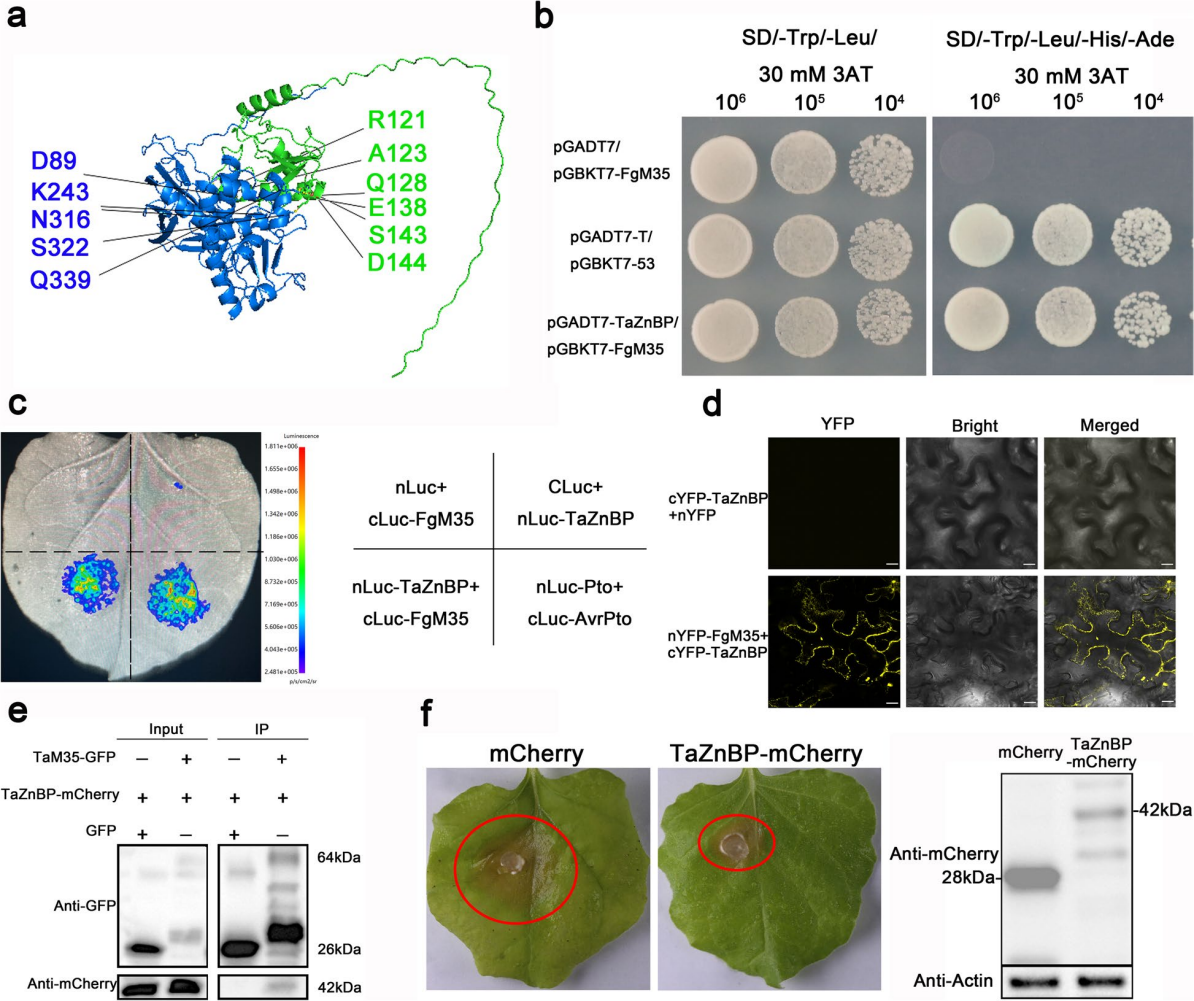
Verification of the interaction between FgM35 and TaZnBP and preliminary validation of TaZnBP’s disease resistance function. **a**, Marking the interaction sites of TaZnBP and FgM35 using PyMOL software. **b**, Using a yeast two-hybrid system with pGADT7-TaZnBP/pGBKT7-FgM35, (pGADT7-T/pGBKT7-53) as positive control, and (pGADT7 /pGBKT7-FgZnBP) as negative control. All combinations were able to grow on SD/-Trp/-Leu lacking media; however, only positive controls pGADT7-T/pGBKT7-53 and pGADT7-TaZnBP/pGBKT7-FgM35 were able to grow on SD/-Trp/-Leu/-His/-Ade quadruple lacking media. **c**, Co-injection of *A. tumefaciens* containing cLuc-FgM35/nLuc-TaZnBP into *N. benthamiana* leaves, with cLuc-AvrPto/nLuc-Pto as positive control, and cLuc-FgM35/nLuc and cLuc/nLuc-TaZnBP as negative controls. Fluorescent signals were detected using a live plant imaging system 48 h post-injection. **d**, Co-injection of *A. tumefaciens* containing cYFP-TaZnBP/nYFP-FgM35 and cYFP-TaZnBP/nYFP into *N. benthamiana* leaves, with fluorescent signals in cells examined under a fluorescence microscope 48 h post-injection. **e**, Co-immunoprecipitation of Egfp-FgM35 and TaZnBP-mCherry expressed in *N. benthamiana*. Total protein was extracted 2 d after infiltration, and GFP immunoprecipitation was performed. **f**, Transient expression of TaZnBP-mCherry in *N. benthamiana*, with mCherry as negative control, followed by inoculation with fresh *Phytophthora infestans* sporangia two days after expression and observation and photography three days post-inoculation, accompanied by Western blot analysis of mCherry and TaZnBP-mCherry protein expression levels

Numerous candidate target proteins for FgM35 were identified through screening the wheat yeast cDNA library. Using Alphafold2, we predicted the protein structures of these candidates and downloaded the corresponding PDB files. With the aid of HADDOCK 2.4 (https://wenmr.science.uu.nl/haddock2.4/), we conducted protein–protein interaction predictions for the candidate targets. Among the many candidates, TaZnBP was selected, and the interaction sites between FgM35 and TaZnBP were annotated using PyMOL software (PyMOL 2.2.3, Schrödinger). See Fig. 7a for the results.

To further validate the interaction between FgM35 and TaZnBP, we co-transformed the pGADT7-TaZnBP and pGBKT7-FgM35 vectors into Y2H yeast competent cells for yeast two-hybrid assays. The positive control consisted of pGADT7-T/pGBKT7-53, and the negative control consisted of pGADT7/pGBKT7-FgZnBP. Single colonies grown on SD/-Trp/-Leu deficient medium were diluted in gradients of 10^6^, 10^5^, and 10^4^ and spotted onto SD/-Trp/-Leu/-His/-Ade/3AT plates. It was observed that all tested yeast strains grew well (Fig. 7b), indicating the interaction between the candidate target protein TaZnBP and the effector protein FgM35 in the yeast two-hybrid system.

*Agrobacterium tumefaciens* containing cLuc-FgM35 and nLuc-TaZnBP vectors were constructed. These were mixed in equal proportions and infiltrated into *N. benthamiana* leaves using *A. tumefaciens*-mediated infiltration. After 36 h of infiltration, the infiltrated leaves were examined using a plant in vivo molecular imaging system. See Fig. 7c for the results. The (cLuc-FgM35/nLuc-TaZnBP) combination exhibited fluorescence similar to that of the positive control, while the negative control showed no fluorescence, indicating the interaction between FgM35 and TaZnBP.

*A. tumefaciens* containing the cYFP-FgM35 and nYFP-TaZnBP vectors were constructed. These were mixed in equal proportions and infiltrated into *N. benthamiana* leaves using *A. tumefaciens*-mediated infiltration. After two days of infiltration, the infected leaves were observed using laser confocal microscopy to detect YFP fluorescence. The (cYFP-TaZnBP/nYFP) combination served as the negative control. See Fig. 7d for the results. Under laser confocal microscopy, no fluorescence signal was detected in the negative control, while the (cYFP-FgM35/nYFP-TaZnBP) combination exhibited YFP fluorescence, indicating the interaction between FgM35 and TaZnBP.

The interaction between FgM35 and TaZnBP proteins was further validated through Co-IP assay. Recombinant pBin-eGFP-M35 + pich-TaZnBP-mCherry and pBin-eGFP + pich-TaZnBP-mCherry were co-expressed in *N. benthamiana*. Total protein was extracted from infiltrated leaves and subjected to immunoprecipitation (IP) using antibody-coated magnetic beads capable of binding GFP. Detection using anti-GFP and anti-mCherry monoclonal antibodies revealed the presence of pBin-eGFP-M35 and pich-TaZnBP-mCherry in the immunoprecipitates (Fig. 7e). In conclusion, these findings demonstrated the interactions between FgM35 and TaZnBP.

*A. tumefaciens* containing the TaZnBP-mCherry plasmid was injected into *N. benthamiana*, allowing for transient expression of TaZnBP. Two days after the injection, the plants were inoculated with *Sclerotinia sclerotiorum*. Disease symptoms were observed three days later. Furthermore, Western blot analysis was employed to examine the expression levels of mCherry and TaZnBP-mCherry. See Fig. 7f for the results, Compared to results of the control with an empty mCherry vector, the transient expression of TaZnBP-mCherry increased the resistance of *N. benthamiana* to *S. sclerotiorum*, demonstrating the ability of TaZnBP to enhance disease resistance in *N. benthamiana*.

## Discussion

In the natural world, the growth and development of plants are influenced by various factors, including biotic and abiotic stresses. Biotic stresses encompass pathogenic bacteria, fungi, oomycetes, viruses, and more. The coevolution between plants and microorganisms began simultaneously with the first appearance of plants on land (Gehrig et al. 1996). Over millions of years of evolution, an intricate and refined “arms race” has been ongoing between pathogenic microorganisms and plants. In order to invade and propagate, pathogenic microorganisms must penetrate the plant’s defenses and employ their own arsenal to attack (Chen et al. 2023).

During the invasion of host plants by pathogens, the latter secrete effector proteins to counteract the plant’s basal defense reactions. In response, the plants initiate an effector-triggered immunity (ETI) defense reaction. Within the plant, disease resistance proteins recognize the effector proteins secreted by the pathogens, thus triggering the plant’s defense response. Compared to pattern-triggered immunity (PTI), ETI responds more swiftly. It induces an HR response in the plant, leading to cell necrosis, a process of programmed cell death. This containment restricts the pathogens at the infection site, preventing further spread (Jones and Dangl 2006).

According to reports, metalloproteases in fungi are considered important virulence factors (Yike 2011) and are widely distributed in other pathogens (Rawlings et al. 2018). Previous studies have found that the M35 family of metalloproteases possesses a conserved HEXXH motif and belongs to the zinc-dependent metalloprotease class, playing a crucial role in the interaction between pathogens and hosts (Zhang et al. 2021). The protein encoded by the *FgM35* gene in *F. graminearum* is an M35 metalloprotease, known to be dependent on zinc ions to function. These metalloproteases are secreted by many bacteria, fungi, insects and plants, and primarily act as virulence factors in diseases or infections (Overall and Kleifeld 2006). To date, the function of M35 metalloproteases has been studied in only a few fungi. For instance, VdM35-1 from *Verticillium dahliae* was found to induce HR in *N. benthamiana*, depending on signal peptides and the HEXXH domain, suggesting that VdM35-1 can induce plant PTI response and activate plant immunity. However, the significant decrease in pathogenicity upon knocking out *VdM35-1* indicates that it has effector-like functions in addition to its PAMP function (Lv et al. 2022). In *F. oxysporum* f. sp. *cubense* tropical race 4 (Foc TR4), FocM35_1 was found to induce cell death in *N. benthamiana* while suppressing INF1-induced HR, suggesting that the metalloprotease effector FocM35 enhances pathogenicity by suppressing host immunity (Zhang et al. 2021). Our study reported similar findings, as FgM35 suppressed INF1-induced HR and significantly contributed to the pathogenicity of *F. graminearum* (see Fig. 5, 6).

Literature has also revealed that the expression level of metalloprotease *FgM35* in *F. graminearum* is upregulated during infection, as also illustrated by our results in Fig. 1d, signifying its contribution to the process of *F. graminearum* invasion. To further delineate the function of *FgM35* in *F. graminearum*, we conducted gene deletion and complementation experiments on the *FgM35* gene. Phenotypic comparisons between mutants, wild-type strains, and complemented strains shed light on the role of *FgM35* in sexual reproduction. In this regard, the absence of the *FgM35* gene impeded sexual reproduction in *F. graminearum*, as demonstrated in Fig. 3, which aligns with previous findings suggesting that metalloproteases can interact with chitinases. This functionality extends beyond pathogenic invasion and plays a crucial role in the reproductive processes of the pathogen (Ökmen et al. 2018), underscoring the pivotal role of FgM35 in *F. graminearum* intranuclear proliferation. Pertaining to the sensitivity to external pressures, the overall analysis indicated that the *∆FgM35* mutant strain exhibited reduced tolerance to cell wall disruptors and increased tolerance to metal ion stress, as depicted in Fig. 4. This suggests that the activity of the metalloprotease FgM35 was dependent on metal ions and may participate in regulating the stability of the *F. graminearum* cell wall. Similar observations have been made regarding other metalloproteases, whereby their absence affects the pathogen’s tolerance to metal ion stress (Wang et al. 2022). Generally, effector proteins do not contribute much to growth and development, but there are a few effector proteins that play dual functions, such as UmFly1 in *Ustilago maydis*, ΔUmFly1 affects the virulence function as well as cell division (Ökmen B et al. 2018). *F. graminearum* FgFly1 also shows a similar function (Wang et al. 2022).

Zinc, as a vital life element, plays an immeasurable role in the growth, development, and metabolism processes of organisms. More than 300 enzymes or proteins rely on Zn^2+^ as a co-factor to fulfill crucial functions in living organisms (McCall et al. 2000). Whether in plants (Passerini et al. 2006) or microorganisms (Stempniak et al. 1997), Zinc holds substantial significance.

Through experiments involving peptide secretion and overexpression of *FgM35* strains during infection of onion epidermal cells, we confirmed that the signal peptide of FgM35 possessed secretory function. This indicated that during the invasion stage, *F. graminearum* had the ability to secrete FgM35 into wheat to exert its functions. To examine how FgM35 impacts wheat’s resistance to diseases, we conducted screening of FgM35 wheat targets and verified the interaction between FgM35 and TaZnBP (Fig. 7).

In essence, we observed the involvement of *FgM35* in the sexual reproductive process of *F. graminearum*, allowing it to enhance its invasive capability by reducing the plant’s disease resistance. This groundbreaking discovery revolves around the targeting of TaZnBP by FgM35, a zinc-binding protein of crucial importance in plant defense mechanisms. Through the interaction of FgM35 metalloprotease and TaZnBP, we speculate that FgM35 is secreted into wheat, degrading TaZnBP. Subsequently, our future research will build upon these significant findings and focus on delving into the mechanisms of how TaZnBP combats FHB in wheat.

## Conclusion

In summary, our work revealed the indispensable role of FgM35 in the reproductive process and the pathogenicity of *F. graminearum*, and it identified the interaction between FgM35 and TaZnBP as well as the function of TaZnBP. This provides a theoretical basis for further study of the function of metalloproteinases in pathogen-host interactions.

## Materials and methods

### Strains and plant culture conditions

All *F. graminearum* strains (wild-type PH-1, mutant, and complemented strains) were inoculated on potato dextrose agar (PDA) medium and cultured in darkness at 25 °C. *Escherichia coli* DH5α and *A. tumefaciens* GV3101 were cultured in LB (Luria–Bertani) medium at 37 °C or 28 °C. The wheat used in the culture experiment was a medium-resistant FHB variety (Xiaoyan 22), and the growth conditions were natural growth in the field. The *N. benthamiana* used in the experiment was cultured in an incubator at 22 °C and seedlings were used at four weeks of age.

### Generation of FgM35 knockout mutants in *F. graminearum*

In this study, the *FgM35* upstream homologous arm + Hph resistance gene + upstream homologous arm was constructed by double-joint PCR technology (Yu et al. 2004), and the *FgM35* gene was knocked out by polyethylene glycol-mediated protoplast transformation (Proctor et al. 1994). The knockout mutants were screened by PCR and hygromycin resistance, and the knockout results of the gene deletion mutants were determined by PCR detection using the relevant primers (Fu et al. 2022). The primers used in this experiment are shown in Table S1.

### Generation of FgM35 complement transformants

To generate complementary transformants of *ΔFgM35*, we inserted *FgM35* and its own promoter into the vector PYF11-GFP (*XhoI*). PYF11-FgM35-GFP was obtained and then transformed into ΔFgM35 protoplasts by polyethylene glycol-mediated protoplast transformation. All these transformants were screened for G418 resistance and further confirmed by PCR (Zhang et al. 2020).

### Asexual/sexual reproduction assays

In the asexual reproduction test, the activated fresh strains were cultured in liquid carboxymethyl cellulose (CMC) liquid medium for three days using a 6 mm sterile puncher at the edge of the colony, and the conidia were counted according to the previously reported method (Liu et al. 2023). In the conidial germination experiment, the conidia produced by CMC were cultured in Yeast Extract Peptone Dextrose Medium (YEPD) liquid medium for 8 h. Conidia of all strains were observed using an Olympus microscope.

As sexual reproduction requires, the activated strain was inoculated on carrot agar medium, cultured in an incubator at 25 °C for 2 ~ 3 days until the mycelium grew over the entire petri dish, and the mycelium, including the middle of the fungus cake, was scraped with a sterile weighing spoon. 800 μL of 2.5% Tween-20 was applied to the surface of the medium and spread evenly with a sterile weighing spoon. The medium was dried and sealed with a sealing film, then placed in an incubator at: 25 °C, under darkness for 12 h: 12 h under standard conditions of culture, when the surface of the medium grew aerial mycelium. The aerial mycelium was treated with 300 μL 2.5% Tween-20. After the ascospore shell was induced, the morphology of the pressed ascospores and ascospore flowers was observed under a microscope, and the number of ascospore shells was counted (Zhang et al. 2020).

### Vegetative growth and external pressure stress assays

First, the wild-type, mutant, and complemented strains were inoculated onto the center of PDA, complete medium (CM), and minimal medium (MM) solid medium. Inoculate each strain into each medium with three samples of each medium, and cultured at 25 °C. The colony diameters were measured by crossover method every 12 h for three days, and the colony morphology was photographed on the third day. The experiment was repeated three times.

Next, a sterile glass slide was placed in a petri dish, into which the melted PDA medium was poured just until the glass slide remained uncovered. After solidification of the medium, wild-type, mutant and complemented strains were activated by sterile punches. The edge of the colonies were hit and inoculated on the outside of the glass slide, and the temperature was maintained at 25 °C until the mycelium grew on the glass slide. The glass slide was removed and the edge of the mycelium was observed under the microscope and photographed. The experiment was repeated three times.

The wild-type strain, mutant strain, and complemented strain were inoculated on PDA plates containing 0.75 g/L caffeine, 0.02% SDS, 0.2 mg/mL Congo red, 0.05 mg/mL calcofluor white, 2 mM CuSO_4_·5H_2_O, 0.5 M CaCl_2_, 0.4 M MgCl_2_·6H_2_O, 1 M KCl, 1 M NaCl, 15 mM H_2_O_2_, 0.125 ppm tebuconazole, and 0.125 ppm phenamacril. The cells were cultured at 25 °C for three days for phenotypic observation. Mycelial growth inhibition rate (MGIR) was calculated as follows: MGIR = [(N–C)/C] × 100, where C is the diameter of the control colony and N is the diameter of the treated colony. Each experiment was repeated three times independently.

### Determination of pathogenicity and DON production

The wild-type, mutant, and complemented strains were activated. When the colony diameter covered two-thirds of the culture dish, five bacterial dishes were made on the edge of the activated strain colony with a sterilized yellow lance head and placed in 30 mL CMC liquid medium. The culture was shaken at 25 °C and 180 rpm for three days, then the resulting spore solution was filtered with sterilized filter paper through a funnel and 10 μL of spore solution was added to the blood cell counting plate to count the number of spores. The spore concentration was adjusted to 10^5^ conidia/mL. 20 μL of spore solution was inoculated into the middle glume of healthy wheat spikes at the heading and flowering stages, and the locations were marked with a pen. Each strain was inoculated on 30 wheat ears with similar growth vigor. After inoculation, a plastic bag was placed over the wheat ears to trap moisture. After two days, the plastic bag was removed and water was sprayed every day to maintain certain humidity. After half a month, the proportion of severity strains at all levels, average severity, and disease index were calculated. The pathogenic analysis of maize filaments was also carried out. The activated strains were inoculated into the middle of young maize filaments, and cultured at 25 °C for four days to maintain moderate humidity. A vomitoxin ELISA kit (Jiangsu Enzyme Immunity Industry Co., Ltd., China) was used to measure DON production.

### Verification of signal peptide secretion function

The predicted signal peptide fragment shown by SignalP was amplified by PCR and cloned into the *EcoRI* and *XhoI* sites of a pSUC2 vector to express the fusion protein pSUC2-FgM35^sp^. YTK12 competent cells were prepared, and the constructed pSUC2-FgM35^sp^ vector, positive control vector pSUC2-Avr1b, and negative control pSUC2 empty vector were transferred into the YTK12 strain, coated on SD/-Trp solid medium, and cultured at 30 °C for 2 ~ 3 days. The correctly sequenced transformants were shaken at 30 °C in a shaker at 200 rpm. Finally, 100 μL of bacterial liquid was drawn on CMD-W medium and YPRAA medium. If bacteria could grow on CMD-W medium, the transformation was considered successful. Bacteria growing on YPRAA medium indicated that the predicted signal peptide had a secretory function (Xie et al. 2019).

### Yeast two-hybrid assay

The gene DNA fragment was inserted into the pGBKT7 plasmid as a bait vector. According to the yeast protocol manual (Clontech), the bait and prey vector pGADT7-cDNA library (Oebiotech, Shanghai, China) were co-transformed into the yeast strain Y2Hglod, then the positive clones in SD/-Leu-Trp-His-Ade/3-AT medium were selected. In order to confirm the interaction between genes and targets, the recombinant pGBKT7-Bait and pGADT7-Pary were co-transformed into yeast strains and grown in SD/-Leu-Trp-His-Ade/3-AT.

### Bimolecular fluorescence complementation assay

The genes and targets were cloned into bimolecular fluorescence complementation (BiFC) vectors c-YFP and n-YFP, respectively. The constructed BiFC vector was transferred into the competent state of *A. tumefaciens* GV3101 (pSoup-p19) (Shanghai Weidi, China). The correct monoclonals detected by PCR were cultured in LB medium containing kanamycin and rifampicin resistance. The *A. tumefaciens* solution was shaken at 28 °C/200 rpm, then centrifuged at 4000 rpm for 10 min to collect the *A. tumefaciens*. The supernatant was discarded, and 10 mM MgCl_2_ was added to treat the *A. tumefaciens* solution. The c-YFP bacterial solution was mixed with the n-YFP bacterial solution and the P19 bacterial solution according to the OD600 ratio of 0.5: 0.5: 0.3 using acetosyringone (AS) buffer, and then placed in the dark for 2 h and injected into 4-week-old *N. benthamiana*. Fluorescence microscopy was performed after 48–72 h (Hu and Kerppola 2003).

### Luciferase complementation assay

Genes and targets were constructed into pCAMBIA1300-nLuc and pCAMBIA1300-cLuc vectors, respectively. The plasmid was transformed into *A. tumefaciens* GV3101 (pSoup-p19) competent cells, and the culture solution was shaken to an OD value of 1.0. After centrifugation at 700 × g for 5 min, the cells were collected and washed twice with 10 mM MgCl_2_. The OD600 value of each strain was adjusted to 1.0 with 10 mM MgCl_2_ containing 150 μmol/L AS. The two kinds of *A. tumefaciens* strains to be detected were mixed at a volume ratio of 1: 1, so that the OD600 value of each strain was 0.5. The bacterial solution was mixed in an appropriate ratio and injected into *N. benthamiana*. After 48 h, the leaves of *N. benthamiana* with better expression were taken and smeared with luciferase substrate for observation using an in vivo molecular imaging system (CCD imaging system) (Zhao and Zhou 2020).

### Co-Immunoprecipitation (Co-IP) assays

The GV3101 strain of *A. tumefaciens* containing recombinant constructs pBin-eGFP-M35 + pich-TaZnBP-mCherry, pBin-eGFP-M35 + pich-mCherry, and pich-TaZnBP-mCherry + pBin-eGFP were co-infiltrated into the leaves of *N. benthamiana*. Total *N. benthamiana* protein was extracted using cell lysis buffer for Western blotting and IP assays (Beyotime, Shanghai, China) followed by incubation of the protein solution with anti-GFP nanobody agarose beads (AlphaLifeBio, Shenzhen, China) at 4 °C for 4 h. The precipitate attached to the beads was subsequently washed five times with PBS-T buffer (1 × PBS buffer + 0.1% Tween 20). The co-precipitated material was then boiled in SDS protein loading buffer, separated by PAGE-SDS gel electrophoresis, and probed with monoclonal anti-GFP or anti-mCherry antibodies for detection.

### Inoculation experiment of *Sclerotinia sclerotiorum* on *N. benthamiana*

We constructed TaZnBP in the plant expression vector pich-mCherry, transformed it into *A. tumefaciens* and infected four-week-old *N. benthamiana*. After two days of expression in *N. benthamiana*, fresh *S. sclerotiorum* discs were inoculated on the injection wound, and the petiole of *N. benthamiana* was wrapped with sterilized cotton to moisturize. The tray was covered with wet paper towels, and the treated *N. benthamiana* was placed in the tray. The tray was wrapped with plastic wrap to trap moisture and kept at 23 °C for three days for observation.

### RNA extraction and qRT-PCR determination

For RNA extraction, the mycelium was frozen and ground in liquid nitrogen, then RNA extraction was performed using a fungal RNA extraction kit (HUAYUEYANG Biotechnology Co., Ltd, Beijing), according to the manufacturer’s instructions. Each 20 μL reaction used 1 μg of total RNA for reverse transcription, and the reverse transcription kit used All-in-One First-Strand Synthesis MasterMix (with dsDNase) (BestEnzymes Biotech Co., Ltd. JiangSu, China). SYBR Color qRT-PCR was performed in a 20 μL reaction system, including 0.4 μg cDNA, 0.4 μL gene-specific primers (Table S1), 10 μL Taq-HS SYBR® Green qPCR Premix (Rox Separated) (BestEnzymes Biotech Co., Ltd., JiangSu, China), and 5.2 μL ddH_2_O. The qRT-PCR was performed on the ABI QuantStudio™ 6 quantitative real-time PCR system (Applied Biosystems). The relative expression levels of the selected genes were calculated using the 2^−ΔΔCT^ method (Livak and Schmittgen 2001).

### Statistical analysis

Data were analyzed using GraphPad Prism version 8.0.0 for Windows (GraphPad Software, San Diego, California, USA). The data were subjected to the one-way analysis of variance (ANOVA) test. A two-way Student’s t test and Duncan’s multiple range test of least significant difference (LSD) were used when there were more than two means. P values lower than 0.05 and 0.01 were considered statistically significant.

## Supplementary Information

Additional File 1. Supplementary Table 1 Primer sequence table. Fig. S1. Detection of knockout mutants. a.The M lane represents the 5000 bp DNA Marker. In each strain, the first lane was amplified using the identification primers FgM35-ID-F/HPH-R, the second lane with the identification primers HPH-F/FgM35-ID-R, the third lane with the identification primers HPH-F/HPH-R, and the fourth lane with the identification primers FgM35-ID-F/FgM35-ID-R. b. Southern blot of PH-1, ΔFgM35-1 and ΔFgM35-2 using the HPH probe. Fig. S2. Wild-type PH-1 and three FgM35 knockout mutant strains were cultivated for 36 hours at 25°C on PDA plates supplemented with 0.75 g/L caffeine, 0.02% SDS, 200 ppm Congo Red, 0.05 mg/mL CFW, 2.5 mM CuSO4·5H2O, 0.5 M CaCl2, 0.2 M MgCl2·6H2O, and 15 mM H2O2.

## Data Availability

The datasets analyzed during the current study are available from the corresponding author upon reasonable request.

## Abbreviations

FHB: Fusarium head blight
DON: Deoxynivalenol
PDA: Potato dextrose agar
LB: Luria–Bertani
PCR: Polymerase Chain Reaction
CMC: Carboxymethyl cellulose
YEPD: Yeast Extract Peptone Dextrose Medium
CM: Complete medium
MM: Minimal medium
BiFC: Bimolecular fluorescence complementation
Co-IP: Co-Immunoprecipitation
kDa: Kilodaltons
WT: Wild type
RT-qPCR: Reverse Transcription Quantitative Real‑time Polymerase Chain Reaction
OD: Optical delnsity
