# Correction: Zinc metalloprotease FgM35, which targets the wheat zinc-binding protein TaZnBP, contributes to the virulence of Fusarium graminearum

**DOI:** 10.1007/s44154-024-00200-x

**Published:** 2024-11-27

**Authors:** Xin‑tong Wang, Kou‑han Liu, Ying Li, Yan‑yan Ren, Qiang Li, Bao‑tong Wang

**Affiliations:** State Key Laboratory of Crop Stress Resistance and High‑Efciency Production, College of Plant Protection, Northwest A&F University, Yangling, Shannxi, Province 712100 People’s Republic of China

**Correction: Stress Biol 4, 45 (2024)**

**https://doi.org/10.1007/s44154-024-00171-z**

Following publication of the original article (Wang et al. 2024), the authors reported an error in Fig. 4a, which should be updated from:


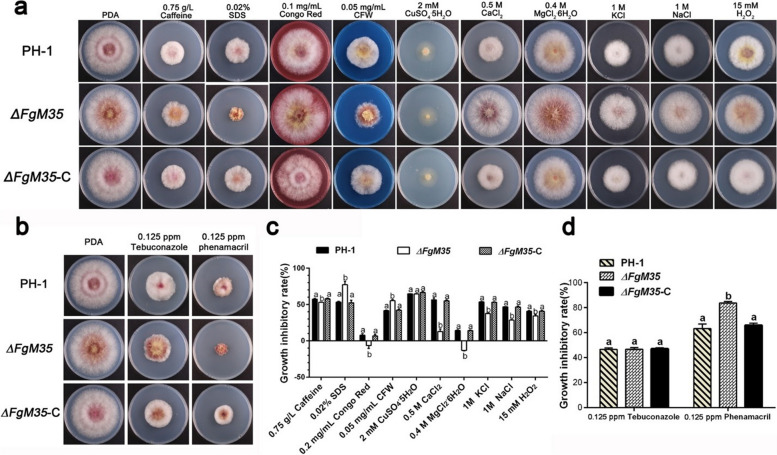


To


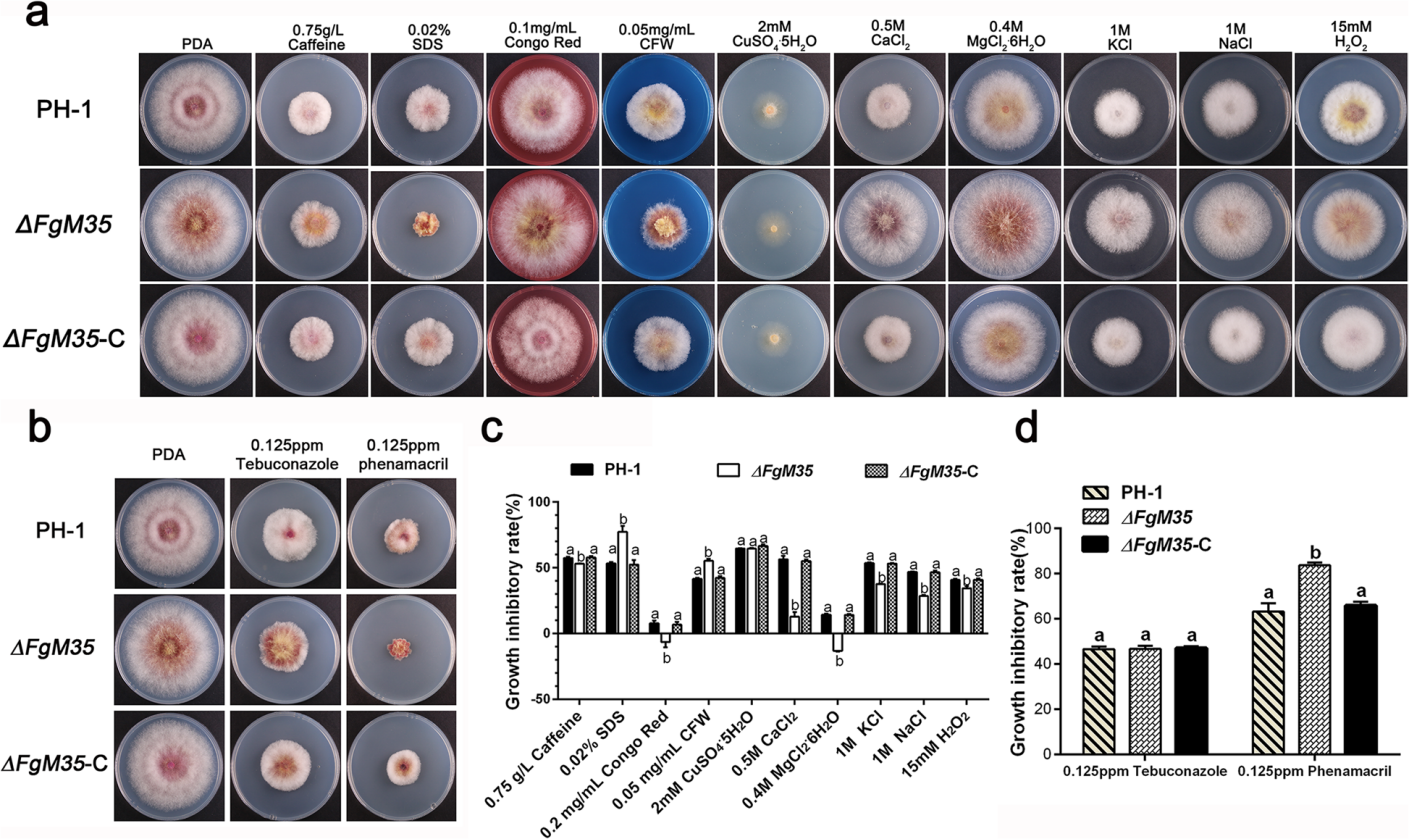


The original article (Wang et al. 2024) has been updated.
