# Supplementary material for: Zinc metalloprotease FgM35, which targets the wheat zinc-binding protein TaZnBP, contributes to the virulence of Fusarium graminearum
Source: Stress Biol. 2024 Oct 30;4(1):45. doi: 10.1007/s44154-024-00171-z (PMC11522218; doi:10.1007/s44154-024-00171-z)
Supplement: Supplementary file 1 — Additional File 1. Supplementary Table 1 Primer sequence table. Fig. S1. Detection of knockout mutants. a.The M lane represents the 5000 bp DNA Marker. In each strain, the first lane was amplified using the identification primers FgM35-ID-F/HPH-R, the second lane with the identification primers HPH-F/FgM35-ID-R, the third lane with the identification primers HPH-F/HPH-R, and the fourth lane with the identification primers FgM35-ID-F/FgM35-ID-R. b. Southern blot of PH-1, ΔFgM35-1 and ΔFgM35-2 using the HPH probe. Fig. S2. Wild-type PH-1 and three FgM35 knockout mutant strains were cultivated for 36 hours at 25°C on PDA plates supplemented with 0.75 g/L caffeine, 0.02% SDS, 200 ppm Congo Red, 0.05 mg/mL CFW, 2.5 mM CuSO4·5H2O, 0.5 M CaCl2, 0.2 M MgCl2·6H2O, and 15 mM H2O2. [file 44154_2024_171_MOESM1_ESM.docx]

Supplementary Table 1 Primer sequence table

| Name | Sequence（5’-3’） |
| --- | --- |
| M35-up-F | ATCGCTTTACTCCGTACGGA |
| M35-up-R | CAAAATAGGCATTGATGTGTTGACCTCCAATGAACGATAGACCAGATC |
| M35-down-F | CTCGTCCGAGGGCAAAGGAATAGAGTAGTTGGAGGTCTCGATGTTATT |
| M35-down-R | TAAGGCTAACTTGGACACAG |
| M35-ID-F | GACCTTCACTTGTGTCAAAC |
| M35-ID-R | CGGCCGTCCCATGGTAGCGC |
| np-M35-F | ACTCACTATAGGGCGAATTGGGTACTCAAATTGGTTATCGAAGTGTAGCCCGAGTC |
| np-M35-R | CACCACCCCGGTGAACAGCTCCTCGCCCTTGCTCACACAACTCATGGAAACAGCCT |
| ID-M35-GFP-F | CGACGTGTTCGCCAAGATCA |
| ID-GFP-R | GTCAGCTTGCCGTAGGTGGCA |
| M35-pBin-F | CCCCGGGGTCGACGGATCCATGCCCCTGACTGGTGGGCG |
| M35-pBin-R | CTCTAGTTCATCTAGAGGATCCACAACTCATGGAAACAGCCT |
| pSUC2-M35SP-F | TCCAAGCTCGGAATTTTAATTAAGAATTCATGAAGTGGCTTGCTGCTGG |
| pSUC2-M35SP -R | CGACTCACTATAGGGAGAACCTCGAGCACAGCAGCAGCCAAAGAGG |
| BD-M35-F | ATGGCCATGGAGGCCGAATTCACCATGAAGTGGCTTGCTGCTGG |
| BD-M35-R | CCGCTGCAGGTCGACGGATCCTCAACAACTCATGGAAACAG |
| BD-F | TAATACGACTCACTATAGGG |
| BD-R | TAAGAGTCACTTTAAAATTTGTAT |
| RT-M35-F | CGTCCTATCATCTGGCGTCC |
| RT-M35-R | ACCTATCACCGTTGCAGTCG |
| AD-TaZnBP-F | GCCATGGAGGCCAGTGAATTCACCATGGCGGCCCCGGCCGCGGC |
| AD-TaZnBP-R | ATGCCCACCCGGGTGGAATTCTTAACCAGGAGGCCATTTCA |
| Nluc-ZnBP-F | GAACACGGGGGACGAGCTCGGTACCATGGCGGCCCCGGCCGCGGC |
| Nluc-ZnBP-R | GCCTCAGTCGACGCGTTGTGGATCCACCAGGAGGCCATTTCATCT |
| Cluc-M35-F | CTCGTACGCGTCCCGGGGCGGTACCATGAAGTGGCTTGCTGCTGG |
| Cluc-M35-R | GCCTCAGTCGACGCGTTGTGGATCCACAACTCATGGAAACAGCCT |
| mCherry-ZnBP-F | CATTTACAATTATCGATATGGCGGCCCCGGCCGCGGC |
| mCherry-ZnBP-R | CTCACCCTAGGACTAGTACCAGGAGGCCATTTCATCT |
| NYFP-M35-F | TCTGAGGAGGATCTTCCTAGGATGCCCCTGACTGGTGGGCG |
| NYFP- M35-R | GGGAGGCCTGGATCGACTAGTACAACTCATGGAAACAGCCT |
| CYFP -ZnBP-F | CTAGTCGACTCTAGCCTCGAGATGGCGGCCCCGGCCGCGGC |
| CYFP -ZnBP-R | ATCGTATGGGTACATCCTAGGACCAGGAGGCCATTTCATCT |
| HPH-F | GGAGGTCAACACATCAATGCCTATT |
| HPH-R | CTACTCTATTCCTTTGCCCT |
| TRI4-F | ACGTGTGGCTACTCAGGAGA |
| TRI4-R | TGGAATTGCCTTGGGGTA |
| TRI6-F | AAATGCCCATTCCCTAGTTG |
| TRI6-R | ATCTCGCATGTTATCCACCCT |
| TRI7-F | TACCGTCGTCTTCAAAACCA |
| TRI7-R | ACGCCAATGGTGTTCACAAA |
| TRI10-F | TCCCAACCTTTCAGAGGTTCA |
| TRI10-R | TGATCCGTCAAGTCTTCCCAT |
| TRI12-F | ACGAACAGCACTGCTACGGT |
| TRI12-R | TTCCTGCTTGTGACTCCAAT |

**
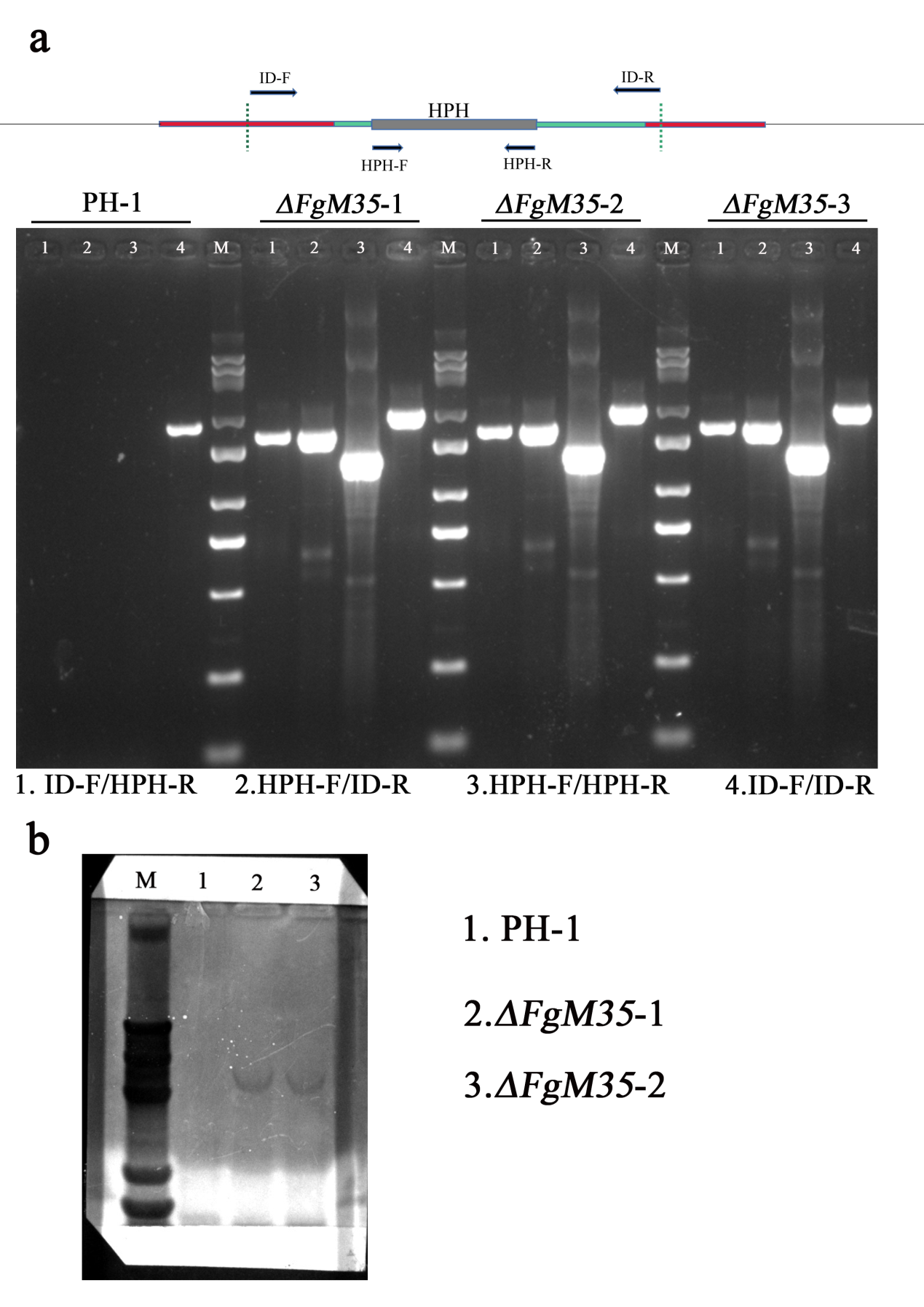
**

**Fig.** **S1**. Detection of knockout mutants. **a**.The M lane represents the 5000 bp DNA Marker. In each strain, the first lane was amplified using the identification primers FgM35-ID-F/HPH-R, the second lane with the identification primers HPH-F/FgM35-ID-R, the third lane with the identification primers HPH-F/HPH-R, and the fourth lane with the identification primers FgM35-ID-F/FgM35-ID-R. b. Southern blot of PH-1, *ΔFgM35*-1 and *ΔFgM35*-2 using the HPH probe.


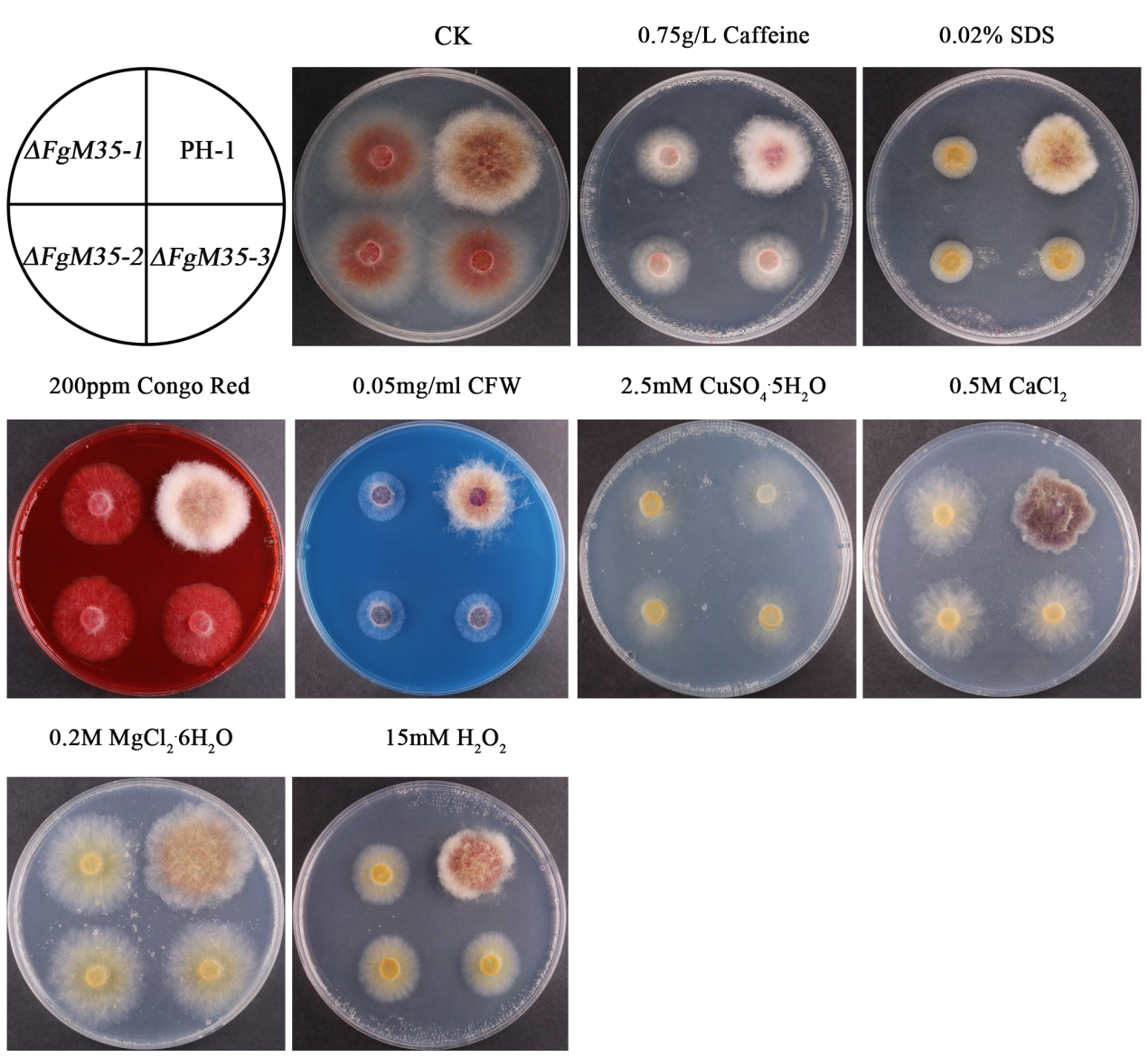


**Fig.** **S2**. Wild-type PH-1 and three *FgM35* knockout mutant strains were cultivated for 36 hours at 25°C on PDA plates supplemented with 0.75 g/L caffeine, 0.02% SDS, 200 ppm Congo Red, 0.05 mg/mL CFW, 2.5 mM CuSO_4_·5H_2_O, 0.5 M CaCl_2_, 0.2 M MgCl_2_·6H_2_O, and 15 mM H_2_O_2_.
